# The Mystique of Late Breakers

**DOI:** 10.1016/j.shj.2022.100097

**Published:** 2022-10-10

**Authors:** Anthony DeMaria

**Affiliations:** Judy and Jack White Chair in Cardiology, Sulpizio Cardiovascular Center, University of California, San Diego, La Jolla, California, USA

I am enroute to the European Society of Cardiology Meetings in Barcelona and planning how best to distribute my time. As is always the case at the large society meetings, there are many simultaneous sessions that I would like to attend. I, therefore, try to plan my schedule in advance. Multiple publications preview the meeting and present their opinion of the likely highlights. Although they do not always agree on those sessions which will be most impactful, all invariably highlight and emphasize the late breaking clinical trials. In fact, nearly all written, online, and video previews feature discussions of the forthcoming Late Breakers. Even medical journals contribute to conveying stature on Late Breakers by featuring them with simultaneous publication. In addition, those programs that review the proceedings of the meeting afterward nearly always focus on the Late Breakers as well. In terms of original research presentations, the Late Breakers have become something of an entity unto themselves.

It is not clear exactly how the entity of a late breaking clinical trial came to such a unique status. Although I hate to admit it, I was the Program Chairman of the American College of Cardiology Scientific Sessions in the 1970s. That year I was contacted 2 ​months before the meeting about a clinical trial that had just been completed and for which results were available. The investigators were anxious to have the results reported at the forthcoming meeting, although they recognized that it was last minute. I indicated to them that the meeting was fully planned, all rooms filled, and all times allotted, but I would discuss it with the committee. The consensus was that the meeting was already set and that trial results would have to wait until the next major meeting. Perhaps the subject matter of the trial influenced the decision; nevertheless, the behavior at that time clearly differed strikingly from that at the present time.

The rationale for the importance and popularity of the Late Breakers is obvious. Of the hundreds of original research presentations or posters presented at the meeting, very few will change or confirm the clinical practice. (Interestingly, the same can probably be said of medical journals that are regularly published.) The meeting presentations contain information that is confirmatory, or extends previous findings, or is hypothesis generating, or is mechanistic, or lays the framework for the ultimate clinical application of the findings, all of which are very important. They may play an important role in guiding practice in specific subspecialties such as imaging or electrophysiology or for very specific disease entities. However, most research presentations do not answer the question of “what should I do differently in my practice when I go home?” In contrast, Late Breakers, if properly selected, contain information that can guide or change clinical practice. The findings are therefore important and of immediate value to cardiovascular practice. In addition, most Late Breakers represent multicenter (often international) efforts that enroll very large patient populations followed for a substantial period of time. Accordingly, the prominence that they play at the meetings is not surprising.

Recognizing the intrinsic value of Late Breakers, it is still worth considering whether the pendulum has overswung. The emphasis on the Late Breakers has cast a significant shadow over the other original research presentations at the meeting. As only a limited number of presentations can be accommodated as Late Breakers, a substantial number of clinically important and relevant studies occur in other sessions. It would be unfortunate if these other data did not attract the attention they deserve. There is also the danger that the hype associated with Late Breakers could reduce the scrutiny with which they were viewed and perhaps give them an excessive degree of credibility. My sense is that giving the designation as a late breaking clinical trial confers a degree of importance and believability over and above other original research presentations. To gain this designation, I have heard that clinical investigators may view the timeline of their studies in terms of the potential meetings at which they could be a Late Breaker. However, detailed critiques of such trials often identify limitations or statistical issues that affect its clinical application. It is uncertain if these limitations come to the attention of the casual observer.

Another consideration regarding late breaking clinical trials is whether the effort to fill sessions with such studies has diluted the qualities that rendered them so impactful. I have the impression that Late Breakers are increasingly divided into those that are very significant and those that are less so. Late Breaker sessions not infrequently contain follow-up of trials, the primary outcome of which had been previously presented. These reports may consist of subgroup analysis or prolonged follow-up of the results. Although such data are undoubtedly useful, in my mind they do not seem to fulfill the same criteria that led to the stature that Late Breakers have come to enjoy. They certainly do not meet the temporal characteristic of being “late” hot off the griddle data. I have seen Late Breakers that presented the Phase 1 trial data based on a surrogate endpoint in less than 3 dozen subjects. Naturally, rapid publication followed. Clearly, there are limitations on the number of very impactful and truly recently completed clinical trials that are available to any meeting. I sometimes wonder if the attraction of having Late Breaking sessions does not play a role in how many studies are selected for meetings.

Having now expressed my reservations about how late breaking clinical trials are handled by meetings, the media, and perhaps even journals, I must admit that they were the first sessions that I viewed in planning my agenda. The best clinical trials clearly alter guidelines, change practice, and improve health care for society. Their importance is unmistakably evidenced by the large attendance that these sessions draw at meetings. It would be regrettable if the stature and credibility conveyed by designation as Late Breaker adversely affected other presentations or became exaggerated or diluted. Perhaps, a little less emphasis and focus on the designation and greater attention to individual studies would be beneficial.

## Funding

The author has no funding to report.

## Disclosure statement

The author reports no conflict of interest.

